# An Introduction to High Intensity Focused Ultrasound: Systematic Review on Principles, Devices, and Clinical Applications

**DOI:** 10.3390/jcm9020460

**Published:** 2020-02-07

**Authors:** Zahra Izadifar, Zohreh Izadifar, Dean Chapman, Paul Babyn

**Affiliations:** 1 Division of Biomedical Engineering, College of Engineering, University of Saskatchewan, Saskatoon, SK S7N 5A9, Canada; 2 Wyss Institute for Biologically Inspired Engineering, Harvard University, Boston, MA 02115, USA; 3 Anatomy & Cell Biology, University of Saskatchewan, Saskatoon, SK S7N 5E5, Canada; 4 Department of Medical Imaging, Royal University Hospital, Saskatoon, SK S7N 0W8, Canada

**Keywords:** high intensity focused ultrasound, clinical device, principle, application

## Abstract

Ultrasound can penetrate deep into tissues and interact with human tissue via thermal and mechanical mechanisms. The ability to focus an ultrasound beam and its energy onto millimeter-size targets was a significant milestone in the development of therapeutic applications of focused ultrasound. Focused ultrasound can be used as a non-invasive thermal ablation technique for tumor treatment and is being developed as an option to standard oncologic therapies. High-intensity focused ultrasound has now been used for clinical treatment of a variety of solid malignant tumors, including those in the pancreas, liver, kidney, bone, prostate, and breast, as well as uterine fibroids and soft-tissue sarcomas. Magnetic resonance imaging and Ultrasound imaging can be combined with high intensity focused ultrasound to provide real-time imaging during ablation. Magnetic resonance guided focused ultrasound represents a novel non-invasive method of treatment that may play an important role as an alternative to open neurosurgical procedures for treatment of a number of brain disorders. This paper briefly reviews the underlying principles of HIFU and presents current applications, outcomes, and complications after treatment. Recent applications of Focused ultrasound for tumor treatment, drug delivery, vessel occlusion, histotripsy, movement disorders, and vascular, oncologic, and psychiatric applications are reviewed, along with clinical challenges and potential future clinical applications of HIFU.

## 1. Introduction

In clinical practice, a variety of different energies have been used for thermal ablation of tissues, including radiofrequency currents, microwaves, laser, thermal conductor sources, and ultrasound. Ultrasound provides several important benefits, such as enabling deeper tissue treatment, improved focus on the target tissue through its small wavelengths, and precise control over the shape and location of energy deposition [1]. Using ultrasound for heating tissues was one of its early clinical applications [2]. It was first recognized when high intensity ultrasound waves used to navigate submarines during World War II, were found to heat up and kill fishes [3]. As early as the 1940s, researchers tried to focus ultrasound waves on body tissues as an alternative to ablative procedures [4].

Over the past two decades, continued advances in imaging, physics, and engineering have enabled precise focusing of ultrasound on deeper targets in the body. High intensity focused ultrasound (HIFU) is one of the more active research areas among non-ionizing ablation methods; such as lasers and microwaves. HIFU treatment is usually guided, assessed, and monitored by either magnetic resonance imaging (MRI) or ultrasound imaging [5]. Recently, high-intensity focused ultrasound (HIFU) and magnetic resonance-guided focused ultrasound (MRgFUS) have proven effective as non-invasive ablation modalities for soft tissues. These methods have now been used to treat thousands of patients globally [6,7,8], with MRgFUS being proposed as an alternative to a wide range of surgical procedures.

The key to HIFU treatment is that the energy delivered is sufficient to increase the tissue temperature to a cytotoxic level very quickly so that the tissue vasculature does not affect the extent of cell killing. Heat coagulation by HIFU is desired for cell reaction with chronic inflammation, and histological signs of fat necrosis in the surrounding normal fatty tissue [9]. Large blood vessels seem less vulnerable to HIFU damage compared to tumor tissues. This is likely due to dissipation of the thermal energy from the vessel wall by the blood flow, which results in safe ablation of the tumor. Deadly complications may also develop if any vital blood vessels are damaged during ablation. This is important when surgical resection of a tumor is contraindicated and ultrasound ablation may be dangerous because of close proximity to major blood vessels. [9]

This review aims to provide an introduction to the physical principles of HIFU, including its heating and mechanical (cavitation) effects in the body, along with a brief overview of the current clinical therapeutic aspects of HIFU.

## 2. Principles behind HIFU

**Figure 1:**
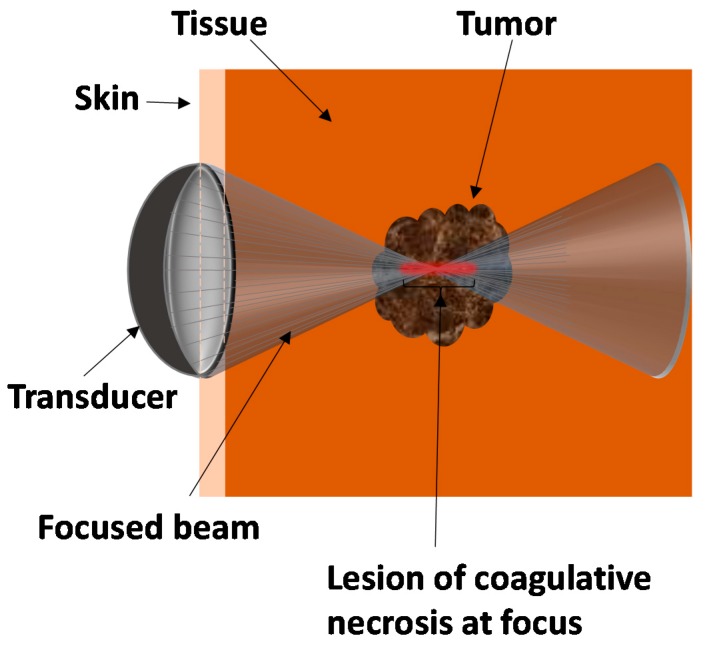
Overview schematic of high-intensity focused ultrasound for tumor therapy.

HIFU beam can pass through overlying skin and tissues without harm, and focus on a localized area with an upper size limit of approximately 3–4 cm in diameter for tumors. Figure 1 shows schematic of a HIFU transducer with focused beam on a tumor. HIFU produces a focused ultrasound beam that passes through the overlying skin and tissues to necrose a localized region (tumor), which may lie deep within the tissues. The affected area at the focal point of the beam leads to lesion coagulative necrosis and is shown in red in Figure 1. When the tumor is ablated, a very sharp boundary between dead and live cells are created [9]. The boundary width between totally disrupted cells and normal tissue is no more than 50 µm [10].

The basic principles underlying the tissue damage from HIFU are tissue coagulative thermal necrosis due to the absorption of ultrasound energy during tissue transmission (thermal effect) and ultrasound-induced cavitation damage [9]. The heat generated by HIFU can result in a rapid rise in temperature in the exposed tissue to more than 60 °C, which leads to immediate and irreversible cell death in most tissues when it lasts longer than 1 s [9]. The highly focused ultrasound beam results in very high intensity at the focal point of the beam within a small volume of about 1 mm in diameter and about 10 mm in length [9], which minimizes potential damage to tissues outside the focal region. Thermal tissue damage due to high temperature exposure is dependent almost linearly on the length of the exposure time and exponentially on the increase in temperature [11].

Another mechanism involved in HIFU ablation is mechanical effect. This mechanical effect, including cavitation, only occurs with high intensity acoustic pulses [9]. Cavitation can generate very high pressures and temperatures, high shear stress, and create microstreaming jets of liquid that can cause pitting of the cell wall. If the medium is mostly liquid and can freely move, then liquid movement can lead to the production of microscopic streaming, which can cause cell apoptosis [12]. The nuclei of these apoptotic cells are self-destructed with degradation of deoxyribonucleic acid (DNA) by endonucleases.

## 3. Ultrasound Beam Delivery System

**Figure 2:**
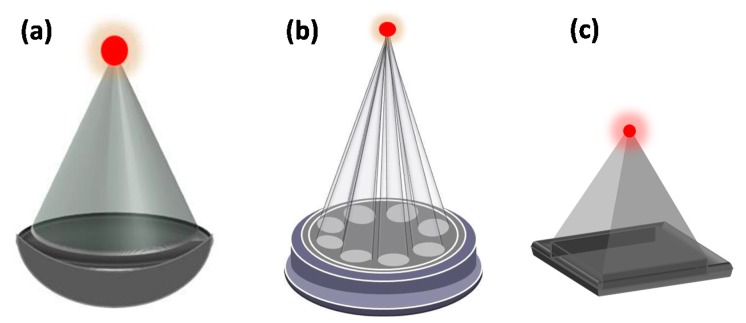
(**a**) Schematic of a concave focusing transducer and (**b**) an arranged multiple piston transducer or the atruncated surface of a spherical bowl (**c**) fully populated phased array.

Overall, HIFU equipment consists of two main components. The first is a piezoelectric ultrasound transducer that is used to deliver the therapeutic ultrasound beam. The most popular type of transducer used is a concave focusing transducer with a fixed aperture and focal length (Figure 2a). Other types of transducers include phased array transducers which comprise multiple piston transducers that are arranged on the truncated surface of a spherical bowl (Figure 2b) or a flat transducer/fully populated phased array (Figure 2c) (e.g., Model-JC HIFU system, Chongqing HAIFU™ Company, Chongqing, China).The mechanical movement of the transducer determines the position of the focal point, with electronic steering of the ultrasound beam allowing fine control of the focal spot location.

## 4. Ultrasound Guidance Modalities

**Figure 3:**
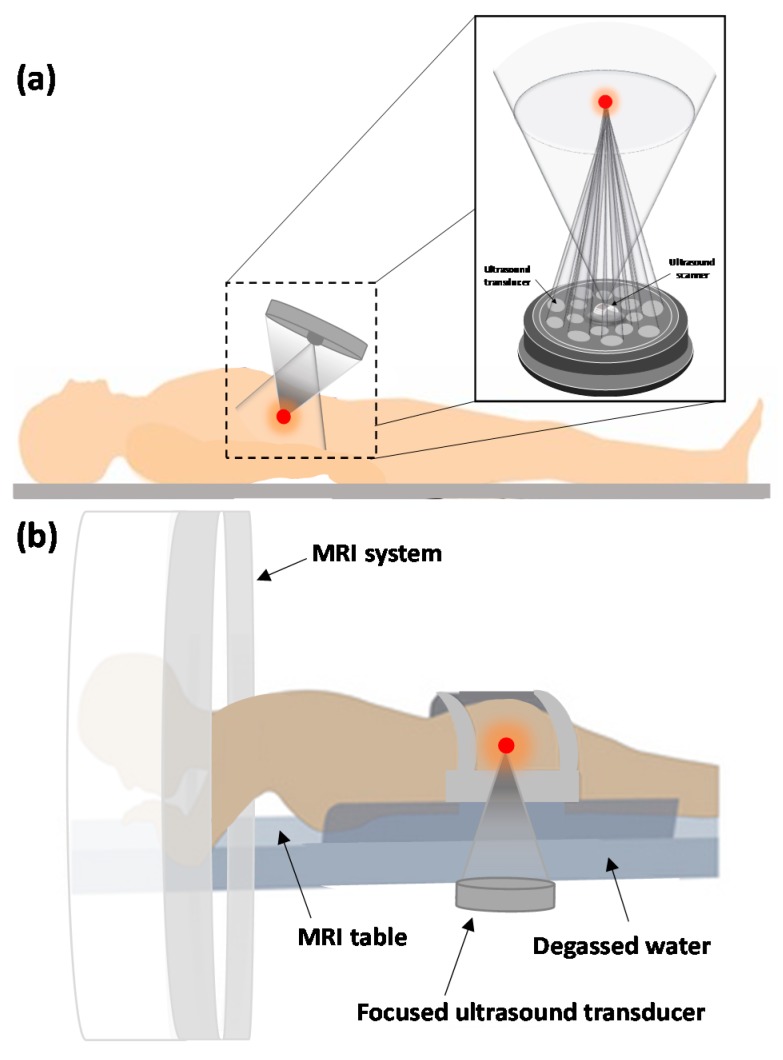
Schematic of (**a**) the structure of an extracorporeal high intensity focused ultrasound (HIFU) transducer, including both imaging and therapy probes, depicting an ultrasound-guided technique on a patient and (**b**) magnetic resonance-guided extracorporeal focused ultrasound system treatment technique.

The second major component of HIFU is the imaging modality used for guidance. Real-time imaging during therapeutic procedure is essential to ensure the safety and efficiency of the treatment. The imaging modalities that have been used for monitoring treatment are sonography and Magnetic Resonance Imaging (MRI). Figure 3a,b, respectively, show schematics of typical ultrasound- and MRI-guided focused ultrasound (USgFUS and MRgFUS) systems applied to the target through the skin for extracorporeal shock wave therapy (ESWT), and HIFU.

### 4.1. MRI

MRI with its high anatomical resolution and sensitivity for tumor detection offers accurate planning of the tissue to be targeted and treated. In addition, MR thermometry enables calculation of the thermal dose and a superimposed representation of the anatomical image of the area where the temperature reaches cytotoxic levels. It provides closed-loop control of energy deposition with a temperature accuracy of 1 °C, spatial resolution of 1 mm, and temporal resolution of 1 s during HIFU treatment [9]. Within seconds of HIFU exposure, MRI can provide temperature data and is superior to sonography for obese patients [13] as it is not limited by fat tissue [14]. However, MRI is expensive, labor-intensive, and its temporal and spatial effects can lead to underestimation of temperature. MRgFUS is good for measuring the temperature that is temporally generated in the tissue, but not for measuring the tissue mortal thermal dose [14].

### 4.2. Sonography

Compared to MRgFUS, ultrasound imaging is more convenient and mechanically compatible, and provides the same form of energy for image guidance as used for therapy. It provides the benefit of verifying the acoustic window with sonography in real time, which means that if the target region is not visualized by ultrasound imaging before and during HIFU therapy, then it is unlikely that HIFU therapy will be effective in that specific region. The ablated target region is not visualized on standard B-mode images unless the gas bubbles produced within the focal zone appear as hyperechoic spots in the image [9]. USgFUS is good for pre-procedural positioning of the target tumor, but not for intra-procedural evaluation of therapeutic boundaries [9].

## 5. Accessibility of the Tissue to Ultrasound

**Figure 4:**
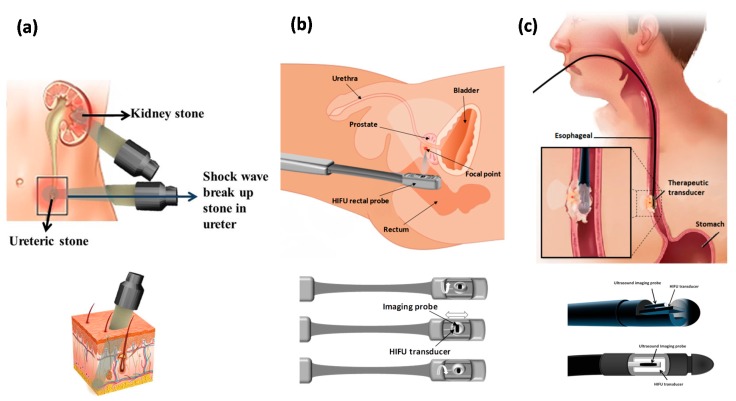
Schematic of high-intensity focused ultrasound applications in (**a**) lithotripsy; with an extracorporeal ultrasound transducer (**b**) prostate cancer; with a typical transrectal ultrasound transducer for prostate cancer treatment with both therapy and imaging transducers incorporated into the head of the transducer probe (**c**) and esophageal cancer; the front and side view of the head of the interstitial transducer used for the treatment of esophageal tumors.

There are three different ways to apply HIFU to the human body based on the accessibility of the targeted organ to ultrasound. When the organ is readily accessible, such as the kidney, HIFU is applied through an acoustic window on the skin by external or extracorporeal transducers (Figure 4a). In other cases like prostate cancer, however, a transducer may need to be inserted into the body (transrectal transducer) (Figure 4b). Interstitial probes are being developed for the treatment of biliary ductal and esophageal tumors and are inserted into the body through the mouth and placed close to the tumor (Figure 4c). Because an extracorporeal device is used to distribute the incident energy over a large skin area, the device has a wide aperture and long focal length to decrease the acoustic intensity at the entry site of the wave site to avoid skin burn. The device requires coupling the acoustic energy to the skin surface via a coupling gel or water balloon, with an appropriate window entry site on the skin so that the propagated focused beam is not interrupted by intervening gas.

Transrectal and interstitial transducers usually operate at higher frequencies and lower power so they can be applied from smaller distances to the target area. The devices developed for transrectal use have combined therapy and imaging transducers incorporated into the head of the transducer probe with a fixed but adjustable focal point that can be mechanically moved to treat a larger tissue volume (Figure 4b). Prostate ablation is performed by creating adjacent lesions side by side and the ultrasound power is altered to adjust the lesion length. For thick prostates, deep lesions are achieved either by making lesions in two layers or by using a longer focal length [9]. For an interstitial transducer, instead of focusing the probe a plane transducer is usually applied and coagulation of the volume is achieved by rotating the probe [15]. With the probe in place, 360° of rotation can be achieved under fluoroscopic or MRI guidance and then the transducer can be repositioned and another adjacent ring of ablation can be produced. This device can also be used for biliary and esophageal tumors or bloodless partial nephrectomy [9]. Interstitial devices can be derived from percutaneous, laparoscopic, or catheter-based ultrasound devices. Catheter-based ultrasound devices can be placed within or adjacent to the target volume directly to treat and coagulate a large volume of the target area, or they can be used as endoluminal and endovascular cardiac devices. The exposure time for catheter-based ultrasound devices is typically 10–30 minutes and the procedure is more invasive compared to external HIFU; however, this method has better energy localization [16]. Catheter-based ultrasound devices are under development for future clinical use for thermal therapy of cancer and benign conditions in the prostate, uterus (fibroids), liver, and bone.

Depending on the geometric size and acoustic parameters of the transducers applied in a HIFU system, the beam size of a −6 dB HIFU system at its focal region is typically 1–3 mm in width and approximately 10 mm in length [9]. However, a 1 cm cancerous tumor is detectable and treatable with HIFU. The concern for inhomogeneity of tissue in abdominal-pelvic (such as in uterine fibroids and renal tumors) or transcranial usage that may cause distortion of the focal beam or a drop in focusing ability in deep-seated tissues is solved by application of a phase correction procedure in the HIFU system, as is done with ultrasound imaging systems [9]. When a larger volume needs to be targeted for ablation, the transducers applied in the HIFU system are mechanically or electronically moved in discrete steps and fired at each point until the result is a confluent regions of cell killing. 

Overall, the therapeutic ultrasound frequency depends on the application-specific treatment depth and the desired rate of heating required for treatment. Higher frequencies have lower penetration depths while lower frequencies have higher penetration depths. Frequencies as low as 0.5 MHz have been used for deep treatments (such as transcranial applications) or high absorption situations and as high as 8 MHz for superficial treatments (including prostate applications) [17]. Frequencies close to 1 MHz have been found to be the most useful for heat deposition [9].

## 6. HIFU Analysis

### 6.1. Benefits

Many benefits justify further exploration of HIFU for additional future clinical applications: HIFU ablation results in reduced toxicity compared with other ablation techniques; it is non-invasive and causes minimal pain; it is a low-cost procedure compared with surgery; less anesthesia involvement and suitable for patients at high surgical risk; it leaves no scars on the patient; lower infection risk; recovery is faster compared with traditional surgery techniques; any bleeding that occurs during the procedure can generally be stopped by ultrasound; it has excellent repeatability as there is no dose limit;, there is no exposure to ionizing radiation due to being guided by MRI or diagnostic ultrasound as opposed to X-ray imaging, precise energy delivery to a targeted point in soft tissue without affecting the skin integrity; system maintenance costs are low; it causes very limited side effects to normal surrounding tissues [9]; patient comfort and safety are maximized; undisturbed real-time visualization can occur during the procedure; and the technique is precise and easy to apply.

### 6.2. Limitations and Complications

In terms of limitations, HIFU treatment is sensitive to patient movement, and near-field heating, and the treatment time can be as long as several hours. When an extracorporeal device is used to distribute the incident energy over a large skin area, the device has a wide aperture and long focal length to decrease the acoustic intensity at the wave site to avoid skin burn. However, severe full skin burns following extracorporeal shock wave lithotripsy (ESWL) for renal calculi [18] or second-degree burns after shock wave lithotripsy [19] have been reported. Also, some patients face post-procedure side effects, such as pain, vomiting, and wounds on their skin. For targets lying within the breast, abdomen, brain, or limbs, an extracorporeal HIFU device is usually employed. During HIFU applications, undesired tissue injury, unwanted burns, and pain can occur as significant ultrasound energy is delivered to a localized area of tissue [20]. In addition, HIFU can rarely cause vasospasm and hemorrhaging when concomitant cavitation is also generated in the tissue [21], impotence and incontinence during prostate cancer treatment [22], or creation of an atrial-esophageal fistula during atrial fibrillation treatment [23]. Furthermore, fistula formation and rib necrosis with delayed rib fracture [24] are also considered to be serious complications that can occur following hepatic and pancreatic cancer treatment [20].

## 7. Clinical Applications of HIFU

HIFU has been used to treat a variety of solid malignant and benign tumors. HIFU has the advantage of being completely non-invasive, extracorporeal, and non-ionizing modality compared to conventional cancer treatment methods such as chemotherapy, radiotherapy, and open surgery. It is also considered as the only non-invasive technique for both primary solid tumors and metastatic disease treatment. Non tumorous conditions such as prostate hypertrophy have also been treated using HIFU technique. Here we discuss the most frequently used clinical applications of HIFU.

### 7.1. Malignant Tumors

#### 7.1.1. Liver

Hepatocellular carcinoma (HHC), is one of the most common and often one of the most difficult to treat liver cancer, especially when multicentric. This can make the outcome of surgical resection poor, with high risk of tumor recurrence [9]. Extracorporeal HIFU has enabled selective ablation of distributed liver tumor nodules. Wu et al., reported treating 68 malignant liver patients with HIFU tumor therapy device (Model JC, Chongqing Haifu, Chongqing, China), and observing coagulative necrosis and damaged tumor blood vessels, or complete tumor disappearance in the targeted areas [25]. In 30 of the treated patients, formal surgical resection following the HIFU resulted in total tumor ablation [25]. Further application of the HIFU device in a series of 474 [26] and 100 patients [27] indicated symptomatic improvement (pain and lethargy) in 87% of patients [27]. A UK-based study (Royal Marsden Hospital, London, UK) reported HIFU treatment of 69 liver cancer patients without need for local anesthesia or sedation [28]. HIFU has also been used in combination with other treatment methods, such as transcatheter arterial chemoembolization technique (TACE), with demonstrated longer survival outcomes compared to TACE alone [29]. Overall, HIFU has been clinically demonstrated to prolong survival period and quality of life in liver cancer patients.

#### 7.1.2. Breast

HIFU can be an effective nonsurgical technique for breast cancer treatment particularly for high-surgical-risk patients and breast-conservation therapy. This is because of its local tumor necrosis effect, lessened requirement for anesthesia [30], shorter recovery time, lower infection risk, and absence of scar formation or compromised skin integrity [31]. HIFU has been applied for treating different breast cancers including invasive lobular carcinoma, ductal carcinoma, and mucinous adenocarcinoma [30,31,32,33,34,35,36,37,38] with reported coagulation necrosis rates of 88-100% [32,33,34,36,37] in the treated breast volume. A 5-year follow-up study indicated a disease-free survival rate of 95%, recurrence-free survival rate of 89%, and 90% reduction in tumor size in treated patients [34]. Reported studies have mainly used HIFU Model JC device [26,32,36], ExAblate 2000 unit (InSightec Ltd., Haifa, Israel) [31,35,37], or custom-made HIFU systems [30,38] at frequencies of 1.5 to 1.7 MHz [30,38]. Following HIFU treatments, local mammary edema, minimal to very few skin burns and minor adverse events have been reported in treated patients [31,33,35,37]. The inability to assess the status of treated margins, due to the lack of pathological specimen, and the need for imaging-based post-procedure assessments, rather than conventional histopathology, are current limitations [9]. Nevertheless, HIFU treatment can effectively induce tumor destruction and loss of propagation activity in breast tumors [39].

#### 7.1.3. Prostate Cancer

Prostate cancer treatment trials using trans-rectal HIFU has shown promise over the past decade in more than 100 sites across the world (Europe, USA, and Asia). Different studies have reported significant drop in prostate-specific antigen (PSA) (to ≤0.2 ng/mL) and promising survival rate in patients [9,40,41,42]. Follow-up studies 2–5 years post HIFU-treatments indicated stable low-levels of PSA and 60–90% negative biopsy rate [41,42]. Clinical HIFU has increased the control rates for treated prostate cancers from 50% (at 8 months) in the early days [43] to 90% in more recent trials [9,41,43]. In addition to focal HIFU treatments, whole-gland treatments have also been used for prostate cancer ablation with a resultant 17 to 35% decrease in tumor incidence, and >90% decrease in tumor volume [44]. For advanced prostate cancers, US hyperthermia has been delivered with interstitial/catheter-based ultrasound applicators combined with high dose brachytherapy [45]. The two commercially available therapy systems are Ablatherm® (EDAP-Technomed, Lyon, France) and the Sonablate®500 (Focus Surgery, Indianapolis, IN, USA), which use endorectal probes containing both the therapy transducers and ultrasound imager. The transducer is designed to move longitudinally and rotationally 180° along and around the probe axis, respectively, providing consecutive focal lesions and complete coverage of tumor volume (Figure 4b).

HIFU has been shown to be a safe technique with minimal side effects; however, some uncommonly encountered complications have been reported including urinary retention, infection, incontinence, urethral stenosis, impotence, rectal fistulas, and chronic pain [46], which have higher rates of occurrence in repeated versus single HIFU treatments [47,48]. Transurethral resection of the prostate before HIFU treatment have been reported to mitigate urinary retention [47,48] and significantly reduces indwelling catheter-required time from 40 to 7 days [41]. Overall, HIFU treatment of prostate cancer is a promising method and particularly suitable for obese patients, men over 65 years of age, or those who are not surgical candidates [49].

#### 7.1.4. Kidney

When renal tumors are small in size, non-invasive HIFU ablation therapy is an attractive alternative method compared to total or partial nephrectomy. The few clinical studies that have examined HIFU for ablation of kidney cancer tumors have reported promising outcomes of histology-proven irreversible and homogenous damage of treated areas [50], tumor necrosis 12 days post treatment in 67% of 30 patients [51], tumor shrinkage 6 month following treatment [51,52], and immediate pain relief in 90% of 13 patients [53]. Devices employed for kidney tumor treatments include extracorporeal HIFU devices (JC-Model devices C-Model devices [51,53]), a prototype focused transducer system (Storz Medical, Tägerwilen, Switzerland [52]), and a laparoscopic HIFU system (Sonatherm1 device (Misonix Inc, Farmingdale, NY, USA) [54]. Studies have reported using HIFU at 1–4 MHz frequency both non-invasively guided by imaging transducer [51], invasviely through laparascopic probe to make direct contact with the tumor [50]; procedures performed under general or epidural anesthesia. Although HIFU has been reported to be successful for treatment of lower renal pole tumors [51,52], it may be unsuccessful for tumors located at the upper renal pole due to energy absorption of the beam by interposed ribs [52].

#### 7.1.5. Esophagus

Esophagus cancer is often identified as a small localized intraluminal squamous-cell carcinoma. Common treatment methods involve surgery and chemotherapy with or without radiation therapy. These methods have overall poor outcomes; with 5-year survival rates of 13–18% [55]. Clinical HIFU tretament of esophageal tumor was first reported in 2008 performed on 4 patients [56]. Complete tumor necrosis was seen in one patient, with objective tumor response and significant improvement in dysphagia within 15 days in all patients [56]. The HIFU system used was an axial-rotating, interstitial ultrasound ablator probe enabling sectorial or cylindrical tumor volumes treatment, with a specialized transducer for delivering parallelepiped-shaped, high-intensity beam (Figure 4c); both of which are particularly suitable features for esophageal tumors. The HIFU applicator is inserted and moved down the esophagus using a long flexible shaft, and then inserted inside the tumor for ablation at 10 MHz frequency for 10 seconds; single lesion occurred at 10 mm distance from transducer [56]. Overall, the clinical results indicate the potential efficacy of intraluminal HIFU therapy for local esophageal tumors.

#### 7.1.6. Pancreas

Pancreatic cancer is typically detected late with 5-year survival rates of <5%. While surgery is only a possible option for 20% of patients, HIFU is emerging as a potential treatment technique. HIFU has been increasingly used either alone, in combination with chemotherapy (gemcitabine), or as additional therapy after failure of chemo/radio therapy [24,57]. Results of HIFU treatments have shown encouraging results in pancreatic tumor ablation with tumor size decrease, resolved pain in up to 80% of patients, and average survival rate of 12.5% (ranging from 8 month to > 3 years) on populations ranging from 30 to 223 patients [57]. Extracorporeal HIFU devices used for pancreatic treatment include ultrasound-guided Model-JC system (HAIFU, Chongqing, China) [24], HIFUNIT-9000 (Shanghai A&S Sci-Tech Co., Ltd, Shanghai, China) [58], and a FEP-BY system (Yuande Biomedical Engineering Limited Corporation, Beijing, China) [59]. HIFU treatment has been done without anesthesia [60] or with general anesthesia [61] or regional anesthesia [58].

Reports on side effects and complications of the HIFU treatment have been variable. While some studies have reported no complications [57], others have reported subcutaneous fat and vertebral necrosis, pain, transient pancreatitis, pseudocysts, and skin burn in 1.1–71% of patients [24]. One study observed major complications of tumor-duodenal fistulas with severe abdominal pain, duodenal stent, and third-degree skin burn in 3–8.5% of patients and second-degree burn was reported in all treated patients [24]. Overall, HIFU-only treatment has achieved pancreatic tumor average size reduction rate of 50%, and in combination with chemotherapy has achieved overall and partial response rates of 43.6% and 14.6%, respectively [59,62].

#### 7.1.7. Brain

**Figure 5:**
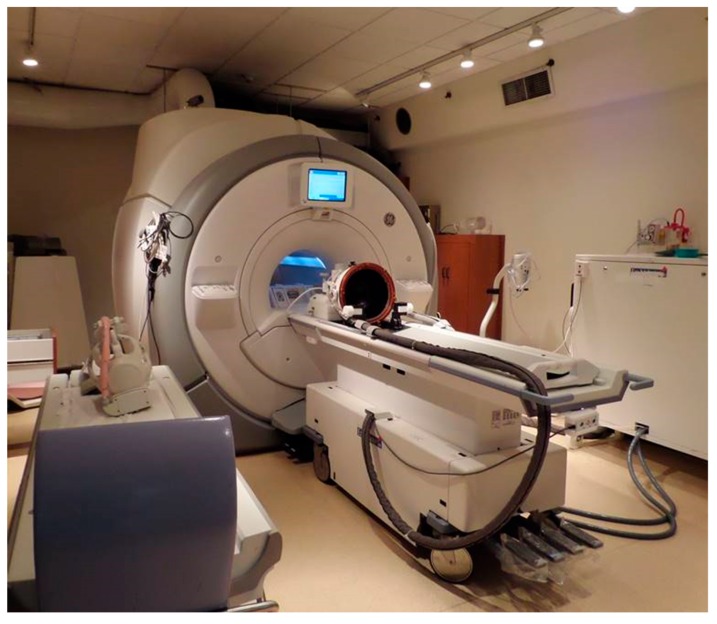
An ExAblate Neuro (InSightec, Haifa, Israel) MRgFUS transducer helmet on an MRI table (located at Sunnybrook hospital, Toronto, Canada).

Glioblastoma is the most common malignant tumor of the central nervous system. It is commonly treated with surgical resection and chemo/radiation therapy. Main challenges in management of brain tumor is the diffuse spread of the tumor throughout the brain and inability of chemotherapy regimen to cross blood brain barrier (BBB) [63]. HIFU, due to its ability to transmit and focus acoustic energy through intact skull and target small areas, has been studied to address these challenges over the past decade with clinical progress mainly in tumor ablation [64]. The first transcranial HIFU surgery on 3 glioblastoma patients showed the feasibility of inducing focal heating in the targeted brain tumor to an overall maximum temperature of 51 °C for 20 seconds sonication time [64]. The patients were treated at acoustic power levels of 800 W (one patient) and 650 W (two patients) [64], which were not enough to achieve brain coagulation and ablation focal thermal threshold of 55 °C [65]. Nevertheless, extrapolation data suggested the feasibility of inducing ablation at 1200 W (55 °C focal peak temperature) without overheating the skull [64,65]. Figure 5 shows a model ExAblate Neuro (InSightec, Haifa, Israel) MRgFUS transducer helmet. Some challenges in brain HIFU treatment include the difficulty of mapping temperature variation in the tumor and cavitation induction and hemorrhage of small capillary vessels at high-intensity sonication [64,65]; HIFU intensity above 4400 Wcm-2 for 1 second can seriously effect blood vessels and cause bleeding [21]. HIFU in immunomodulatory therapy of brain tumor has also been an attractive therapeutic concept. The mechanical cavitation effect of HIFU can induce pro-inflammatory and stress responses, and intra-tumoral immune changes, that when combined with immunotherapy can increase host anti-tumor immune response and overcome glioblastoma multiforme-induced immune evasion [66]. Overall, HIFU appears as a promising technique for brain tumor ablation however further trials are required.

#### 7.1.8. Bone

Reports of HIFU used to treat bone include applications for destruction of tumor microvessels and thrombosis to prevent haematogenous tumor cells dissemination [25], pain palliation of metastatic tumors [67], and treatment of primary bone malignancies [68,69]. HIFU treatment of malignant tumors, either as the sole method or in combination with chemotherapy, in 10–44 patients showed ≤87% survival rate at 10 to 38 months follow-up, complete tumor regression in ≤41.7% of patients, ≥50% tumor volume shrinkage or moderate to partial necrosis/fibrosis in 8.3–33.3% of patients, image-proved inactivation of tumor foci, local tumor reoccurrence or progression in 1–3 patients, complication rate of 18.2%, and metastasis-caused death in 2–5 of stage II and III cancer patients [69]. HIFU treatment of primary bone tumors indicated complete tumor ablation, partial to moderate response, and progression rate of treated patients. HIFU also successfully reduced metastatic bone pain by 69.5–92% at three months post treatment without delaying any post-operative chemotherapy [67]. 

Commercially used HIFU systems in the reported studies are ExAblate 2000 (multielement phased-array transducer, 1.0–1.5 MHz frequency) [67] and Model JC unit (13.5 cm focal length and 0.8 MHz transducers) [68,69]. During treatment, patients may require anesthesia [68,69] or conscious sedation [67]. Mild local pain and edema, first- and second-degree burns [68,69], and third-degree burns requiring further surgical interventions, peripheral nerve damage, bone fracture, ligamentous laxity, epiphysiolysis, and secondary infections [68] were reported as complications of treatment cases in several different studies. Overall, HIFU alone or combined with chemotherapy has been shown as a safe and very effective way of treating malignant bone tumors. The limb-salvaging ability of HIFU (preserving good function in the limbs) make HIFU a promising modality both for tumor treatment and beneficial for revascularization and repair of inactivated bones [9]. It can also be an alternative non-invasive method for palliation of pain in skeletal metastases with several key advantages over other non-invasive treatment modalities [58]. Given the positive results of initial pilot studies, further study is needed [70].

### 7.2. Benign Tumors and Conditions

#### 7.2.1. Uterine Fibroids

Uterine fibroids or uterine leiomyomata are benign smooth muscle tumors of the uterus, fallopian tubes, broad ligament, or cervix, affecting about 25% of women [71]. HIFU is a non-invasive treatment with potential for fertility preservation, and reduced recovery time. Since its approval by the Food and Drug Administration (FDA) in 2004, HIFU has been used for treating more than 2000 patients around the world [9,72]. Clinical trials for treating large fibroid volumes with symptomatic uterine fibroids showed a 10-point decrease in symptom severity score in 79% of treated patients [73], with fibroid volume reductions of 31% after 3 months, 13–33% after 6 months [72,73], and 9.3% after 12 months [74]. These results are based on the FDA guideline of treating only 10% of the fibroid volume, 180 minutes treatment time, and serosa-fibroids distance of at least 15 mm [72]. 2.8 years follow-up study of 138 patients treated with HIFU reported additional undertaken treatments for 19 and 23% of patients (mostly in patients <43 years old) at 36 and 48 months post-treatment, respectively [75].

Commercially available systems used in these studies are ExAblate 2000 (Insightec, Haifa, Israel), a Haifu JM therapeutic system (JM2.5C, Chongqing Haifu Technology Co., Ltd., Chongqing, China), HIFUNIT 9000 (Shanghai Aishen Technology, Shanghai, China), and a custom made mobile HIFU unit (Storz Medical AG, Kreuzlingen, Switzerland). HIFU treatment of large fibroids; ~4 cm in dameter, >45 cm^3^ volume, in less than 15 min has also been demonstrated on excised tumors using an inserted interstitial ultrasound ablator [76]. The HIFU devices are flexible to be directed towards the fibroid tumore while adjacent sensitive strucutres of bowel, skin, and sacral nerve can be spared from acoustic beam exposure [72]. Although clinical outcomes of HIFU is mainly estimated based on questionnaires, long-term follow-ups on fibroid volume reduction, symptom relief, and treated-area apparent diffusion coefficient; a measure of the magnitude of water molecules diffusion within the tissue, remaine to be determind [75]. Overall, HIFU ablation appears to be an effective and safe treatment for symptomatic fibroids particularly for patients unresponsive to medical treamtnets [77].

#### 7.2.2. Breast

Fibroadenomata (FAD) are benign breast lesions typically removed surgically. In addition to conventional surgical removal of the lump or vacuum-assisted mammotomy, other techniques such as HIFU, cryo- or laser ablation have also been used. A comprehensive review of these techniques by Peek et al. indicated that all these ablative techniques are minimally invasive and promising for FAD treatment [78].

#### 7.2.3. Brain Disorders

The ability to focus and target the US beam through the intact skull to areas as small as a couple of millimetres has been a considerable milestone in enabling precise, local ablation of intracranial brain tissue to overcome certain brain disorders [63]. HIFU has been investigated for treating different brain disorders including movement disorders (essential tremor; ET) [79,80], Parkinson’s disease (PD) and Alzheimer’s disease (AD) [81], depression/anxiety and pain syndromes-186 [82,83] [84], epilepsy, thrombolysis/intracerebral hemorrhage (188,189) [82,85,86,87], and cerebrospinal fluid (CSF) diversion [88].

#### 7.2.4. Essential Tremor

Essential tremor (ET) is a movement disorder affects the upper extremities and dominant arm. When severe it is often managed by surgical disruption of the ventral intermediate nucleus of the thalamus; achieved by insertion of the invasive probe and radio frequency (RF) thalamotomy at 75–80 °C or by deep stimulation of the brain that may cause infection and/or hemorrhage [63]. Recent HIFU treatment trials on medically refractory ET patients (4–15 patients) indicated successful thermal ablation of the thalamic target, immediate tremor improvement, 75–89.4% tremor reduction at 1, 3, 6 and 12 months follow-ups, 40% reduction in secondary functional impairment, and gradual evolution of lesions at 1 week and 1 to 3 months follow-ups [79,80,89]. Post HIFU treatment, some adverse effects have been observed in the patients including paraesthesia (in 25–27% of patients), transient sensory, cerebellar, motor, speech abnormalities, mild post-operative balance issues, and development of deep vein thrombosis; however, no serious adverse event have been reported [79,80,89].

#### 7.2.5. Parkinson’s Disease

Parkinson disease is characterized by progressive degeneration of motor neurons. Disruption of key motor nuclei can lead to significant improvement in motor symptoms [90]. HIFU can be a potential non-surgical thermal ablation technique for treating Parkinson’s disease through deep targeting and thermocoagulation of pallidothalamic tract [63]. The first clinical study of this application reported treating 13 chronic and therapy-resistant patients by applying HIFU and stepwise increase of target temperature up to 52–59 °C; 54 °C required for 100% necrosis) [91]. The results indicated higher primary relief; 60.9 versus 7.6%, larger thermocoagulation volumes; 172 versus 83 mm3, and higher global symptom relief at 3-month follow-up; 56.7 versus 22.5%, in group 2 patients compared to group 1, respectively [91]. The results showed no sign of thermal lesion in the follow-up images, no procedure- or device-related neurological side effects, and reported targeting accuracy of <1 mm that demonstrate feasibility [91], safety and accuracy of the HIFU pallidothalamic tractotomy.

#### 7.2.6. Chronic and Non-Malignant Pain

Central- and peripheral-type pain syndromes are challenging conditions to treat especially when common pharmacological medications are ineffective. Destructive procedures can be used to ablate the sensory or affective components; brain and spinal cord in central- and nerves or nerve bundles in periphery-type chronic pain cases [92]. HIFU has been tested for targeting posterior thalamic central lateral nucleus in neuropathic patients and the results indicated 30 to 100% pain relief in 48 hours or at 3 and 12 months post treatment and lesion size of 3–5 mm [83,84]. Potential safety issues reported include small hemorrhage or bleeding complications in the motor thalamus area, possibly caused by cavitation or sonication temperature. These may be avoided by detection of cavitation and the maintenance of sonication temperatures below 60 °C [84].

#### 7.2.7. Benign Prostate Conditions

HIFU treatment of benign prostate conditions presents different problems compared to malignant prostate cancer, since prostate cancer is generally a multi-focal disease. Whole gland HIFU ablation has been more successful in benign tumors [9]. Prostatic hyperplasia has been treated successfully with HIFU by making irreversible lesions in the prostate tissue without any rectal wall damage [93]. However, the long-term outcomes post trans-rectal HIFU therapy were not encouraging [94].

#### 7.2.8. Thyroid

Thyroid nodules have been treated using a variety of different techniques, with surgery still the most common and effective approach. This involves risks and complications of hypocalcemia, transient or permanent recurrent laryngeal nerve palsy, bleeding, postoperative infections [95]. Recently, efficacy of HIFU has been successfully demonstrated for ablation of solid and complex thyroid nodules [96,97,98]. Positive results reported maximum shrinkage with up to 50% reduction in nodule volume at 12 months post-treatment without any change in thyroid function [96,99,100,101] [102]. No major complication or significant collateral damage to neighboring tissues was reported [103]. Reported complications include pain during the procedure, spreading pain toward the neck, scapula, trapezius muscle, or arm [98], mild skin redness, subcutaneous edema [96,97,98], transient vocal cord palsy, and Horner syndrome [99] all of which were not serious and usually disappeared spontaneously within a few days after the treatment [101]. While HIFU is considered an effective technique for thyroid nodule ablation, more studies and data are still required.

#### 7.2.9. Brain

**Figure 6:**
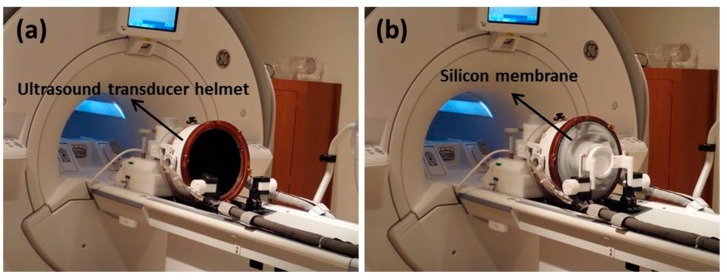
(**a**) The patient lies on the MRI bed and the head is placed inside the phased-array ultrasound transducer helmet. (**b**) The patient’s head is covered with a flexible silicon membrane that is sealed to outer face of the ultrasound transducer helmet. Degassed and chilled water is circulated in the volume between the patient’s head and the transducer to cool down the surface temperature and avoid damage. This water is also used to fill the space between the patient’s head and the transducers to keep the skull bone temperature within a safe range.

The main HIFU device used to deliver medication into human brain for a wide variety of brain diseases and also for scalpel-free surgery of brain tumour is the ExAblate Neuro (InSightec, Haifa, Israel), which consists of a spherical, phased array, multi-element (1000 transducers) helmet that is computer controlled for wave front distortion compensation (Figure 6). Patients usually undergo local anaesthesia for the procedure [63]. The following sections discuss diseases with reported human trials.

#### 7.2.10. Imaging Guided HIFU

Diagnostic imaging systems have been used along with HIFU devices to provide improved safety, therapy navigation, and provide assessment of vascularity or ablation quality during or after treatments. The main reported imaging methods are ultrasound guided FUS (USgFUS) [24,59,62,104] 868585 and magnetic resonance guided FUS (MRgFUS) [105]. Depending on the specific application, imaging and HIFU applicator systems can be physically integrated or separated. Systems such as ExAblate 2000 unite are integrated with MRI and provides planning and real-time thermal mapping and monitoring. This system has been mainly used for brain tumor applications due to the high sensitivity of the target tissue and the need for high precision and effective treatment [84]. MRgUS is suggested to be superior to USgFU with respect to feasibility and efficiency, and the capability to detect deep lesions in the body and to monitor temperature elevation in the treated tissue [106].

## 8. Potential Upcoming HIFU Clinical Applications and Techniques

### 8.1. Vessel Blockage by HIFU

HIFU is emerging as a potential technique for occluding blood vessels in different diseases or conditions such as in arteriovenous malformations to control hemorrhage or in shrinking a solid tumor by blocking/interrupting its blood supply [107]. Further studies/data are required to characterize the HIFU intensity and blood vessel diameter/flow velocity relationship required for successful flow occlusion, and assess its possible long-term adverse effects [9].

### 8.2. Blood-Brain Barrier Disruption

The mechanical disruption ability of HIFU, mediated by cavitation, makes it an attractive technique to locally open the blood brain barrier (BBB); Cavitation bubble-induced BBB disruption can be performed either by very high HIFU exposure, which may cause blood vessel rupture or occlusion [21], or more efficiently by using HIFU+injected microbubbles (ultrasonographic (US) contrast agent) [108]. The later approach can concentrate energy, mediate bioeffects, and open up the BBB within seconds at HIFU power of <0.1% of that required thermal coagulation [108]. Preclinical studies have shown proof-of-concept results generating brief BBB disruption and allowing safe delivery of significant complex and large biologic agents into brain tissue [109,110] The generated opening is generally healed within 6–24 h post-treatment [111]. HIFU BBB disruption trials for human brain tumor treatment are underway at the University of Toronto [63].

### 8.3. Stroke and Thrombolysis

Intracerebral hemorrhage is currently treated with thrombolytics and surgery. HIFU offers the capability to liquefy blood for facilitated aspiration, which can help decrease clot burden and mass effect to avoid craniotomy [63]. Blood clot lysis can be sufficiently achieved through inertial cavitation effect of high-power HIFU with no need for injecting microbubbles [63]; >90% intracerebral hemorrhage clot liquefaction within seconds [82,85,86,87]. Bonow et al [112] has recently hypothesized that transcranial HIFU may have the ability to induce therapeutic cerebral vasodilation and, as a result, may one day be used for treatment of patients with subarachnoid hemorrhage. Furthermore, HIFU appears as a potential technique for clinical treatment of other cerebral ischemic disorders, cerebral vasospasm and other cerebrovascular diseases [112].

### 8.4. Abscesses

Abscesses especially when caused by methicillin-resistant Staphylococcus aureus (MRSA) bacteria may be difficult to treat. A recent feasibility study applied HIFU, at two focal temperatures of 52 and 64 °C, on targeted MRSA-induced abscesses in mice. Post HIFU ablation of 64 °C, significant reduction in bacterial load and abscess external size was observed at day 4 and 10, respectively [113]. No side effect of local neutrophil recruitment, systematic inflammatory response, or open wounds was reported, which indicates the promise of HIFU technique for treatment of localized MRSA-related infections. Extended trials on patients are still needed.

### 8.5. Emerging Focused Ultrasound Techniques

**Figure 7:**
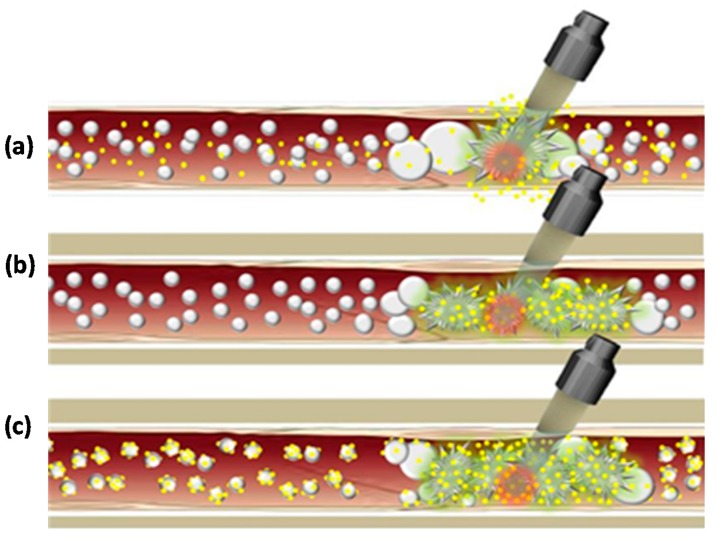
Schematic of different ways of drug delivery utilizing microbubbles. (**a**) Free drug particles (yellow circles) are circulated along with ultrasound microbubbles (grey circles) in vessels, and the effect of ultrasound on growth and burst of microbubbles results in extravasation of drug into adjacent soft tissues. (**b**) Drugs encapsulated inside microbubbles are circulated in the vasculature and microbubbles are ruptured by ultrasound and the transported substances are released into the surrounding targeted tissue. (**c**) Drugs loaded on the external membrane of microbubbles, freely circulated in vessels and then ruptured by ultrasound with the drug being released into the target.

Integrating focused ultrasound with different existing therapeutic technologies can improve clinical outcomes. Figure 7 shows schematic of different ways that drug delivery can be enhanced using ultrasound-microbubble techniques. The advantage of ultrasound-microbubble techniques over other techniques such as nanoparticle or liposome delivery systems is the ability for precise external control [20]

Enhanced skin permeabilization using this technique may replace multiple needle use for medicines such as heparin and insulin or enable diffusion of large molecules (>500 Da) through stratum corneum [114,115]. Focused ultrasound-mediated gene therapy is also a potential application under extensive study [116]. Magnetic resonance-guided focused ultrasound thalamotomy for treatment of ET is another emerging, minimally invasive thermoablation technique for medically refractory ET. MRgFUS thalamotomy leads to sustained tremor reduction for medically refractory ET in the long term [117]. MRgFUS thalamotomy to relieve medication-resistant tremor has been reported as a safe and effective technique in patients with ET, PD, and ET-PD (patients with ET who developed PD in many years later [118,119]. Application of HIFU for neuromodulation has become one of the fastest-growing areas in neuroscience and a new frontier for mental health treatment [120]. Potential usefulness of MRgFUS for obsessive compulsive disorders [121], depression, schizophrenia and anorexia nervosa.

## 9. Conclusions

Both thermal and non-thermal (cavitation) effects play a very important role in all therapeutic applications of ultrasound. The side effects of these two mechanisms of action can be injurious biologically and are therefore avoided in diagnostic applications of ultrasound but can be beneficial in therapeutic applications. The ability to focus the ultrasound beam to a small area a couple of millimeters in size enhances both the thermal and non-thermal effects of ultrasound and results in ablation and necrosis of cells at the applied focal point. This makes ultrasound an excellent non-invasive therapeutic ablation technique for deep-seated targets within the body. HIFU therapy provides a less invasive approach to cancer therapy that minimizes discomfort to the patient and length of hospital stay. Initial studies have demonstrated HIFU to be generally safe and clinically effective and to have high potential clinical acceptance. However, HIFU is still in its infancy and further studies are necessary (especially in the field of oncology and the brain) regarding the long-term medical benefits, technical considerations, and treatment delivery before transition to more widespread use. The range of HIFU applications may expand in the future with improved imaging. MRgFUS is one of the most successful imaging guide approaches. However, there is a need for additional studies with longer-term follow-up.

## References

[1] Lafon C., Melodelima D., Salomir R., Chapelon J.Y. (2007). Interstitial devices for minimally invasive thermal ablation by high-intensity ultrasound. Int. J.Hyperth..

[2] Lehmann J.F. (1953). The biophysical basis of biologic ultrasonic reactions with special reference to ultrasonic therapy. Arch. Phys. Med. Rehabil..

[3] Woo J. (2002). A Short History of the Development of Ultrasound in Obstetrics and Gynecology. http://www.ob-ultrasound.net/history1.html.

[4] Fry F., Ades H., Fry W. (1958). Production of reversible changes in the central nervous system by ultrasound. Science.

[5] Jolesz F.A. (2009). MRI-guided focused ultrasound surgery. Annu. Rev. Med..

[6] Cline H.E., Hynynen K., Watkins R.D., Adams W.J., Schenck J.F., Ettinger R.H., Freund W.R., Vetro J.P., Jolesz F.A. (1995). Focused US system for MR imaging-guided tumor ablation. Radiology.

[7] Hynynen K., Hynynen K., Damianou C., Darkazanli A., Unger E., Schenck J.F. (1993). The feasibility of using MRI to monitor and guide noninvasive ultrasound surgery. Ultrasound Med. Biol..

[8] Hynynen K., Darkazanli A., Unger E., Schenck J. (1993). MRI-guided noninvasive ultrasound surgery. Med. Phys..

[9] Zhou Y.-F. (2011). High intensity focused ultrasound in clinical tumor ablation. World J. Clin. Oncol..

[10] Ter Haar G., Rivens I., Chen L., Riddler S. (1991). High intensity focused ultrasound for the treatment of rat tumours. Phys. Med. Biol..

[11] Dewey W.C. (2009). Arrhenius relationships from the molecule and cell to the clinic. Int. J. Hyperth..

[12] Lagneaux L., de Meulenaer E.C., Delforge A., Dejeneffe M., Massy M., Moerman C., Hannecart B., Canivet Y., Lepeltier M.F., Bron D. (2002). Ultrasonic low-energy treatment: A novel approach to induce apoptosis in human leukemic cells. Exp. Hematol..

[13] Yagel S. (2004). High-intensity focused ultrasound: A revolution in non-invasive ultrasound treatment?. Ultrasound Obstet. Gynecol..

[14] Jolesz F.A., McDannold N. (2008). Current status and future potential of MRI-guided focused ultrasound surgery. J. Magn. Reson. Imaging.

[15] Makin I.R., Mast T.D., Faidi W., Runk M.M., Barthe P.G., Slayton M.H. (2005). Miniaturized ultrasound arrays for interstitial ablation and imaging. Ultrasound Med. Biol..

[16] Salgaonkar V.A., Diederich C.J. (2015). Catheter-based ultrasound technology for image-guided thermal therapy: Current technology and applications. Int. J. Hyperth..

[17] Haar G.T., Coussios C. (2007). High intensity focused ultrasound: Physical principles and devices. Int. J. Hyperth..

[18] Rao S.R., Ballesteros N., Short K.L., Gathani K.K., Ankem M.K. (2014). Extra corporeal shockwave lithotripsy resulting in skin burns—A report of two cases. Int. Braz J. Urol.

[19] Rangarajan S., Mirheydar H., Sur R.L. (2012). Second-Degree Burn after Shock Wave Lithotripsy: An Unusual Complication. BJU Int..

[20] Miller D.L., Smith N.B., Bailey M.R., Czarnota G.J., Hynynen K., Makin I.R. (2012). Overview of therapeutic ultrasound applications and safety considerations. J. Ultrasound Med..

[21] Hynynen K., Chung A.H., Colucci V., Jolesz F.A. (1996). Potential adverse effects of high-intensity focused ultrasound exposure on blood vessels in vivo. Ultrasound Med. Biol..

[22] Rove K.O., Sullivan K.F., Crawford E.D. (2010). High-intensity focused ultrasound: Ready for primetime. Urol. Clin. North Am..

[23] Borchert B., Lawrenz T., Hansky B., Stellbrink C. (2008). Lethal atrioesophageal fistula after pulmonary vein isolation using high-intensity focused ultrasound (HIFU). Heart Rhythm.

[24] Jung S.E., Cho S.H., Jang J.H., Han J.-Y. (2011). High-intensity focused ultrasound ablation in hepatic and pancreatic cancer: Complications. Abdom. Imaging.

[25] Wu F., Chen W.Z., Bai J., Zou J.Z., Wang Z.L., Zhu H., Wang Z.B. (2001). Pathological changes in human malignant carcinoma treated with high-intensity focused ultrasound. Ultrasound Med. Biol..

[26] Wu F., Wang Z.B., Chen W.Z., Wang W., Gui Y., Zhang M., Zheng G., Zhou Y., Xu G., Li M. (2004). Extracorporeal high intensity focused ultrasound ablation in the treatment of 1038 patients with solid carcinomas in China: An overview. Ultrason. Sonochem..

[27] Li C.X., Xu G.L., Jiang Z.Y., Li J.J., Luo G.Y., Shan H.B., Zhang R., Li Y. (2004). Analysis of clinical effect of high-intensity focused ultrasound on liver cancer. World J. Gastroenterol..

[28] Ter Haar G. (2001). Acoustic surgery. Phys. Today.

[29] Wu F., Wang Z.B., Chen W.Z., Zou J.Z., Bai J., Zhu H., Li K.Q., Jin C.B., Xie F.L., Su H.B. (2005). Advanced hepatocellular carcinoma: Treatment with high-intensity focused ultrasound ablation combined with transcatheter arterial embolization. Radiology.

[30] Huber P.E., Jenne J.W., Rastert R., Simiantonakis I., Sinn H.-P., Strittmatter H.-J., von Fournier D., Wannenmacher M.F., Debus J. (2001). A new noninvasive approach in breast cancer therapy using magnetic resonance imaging-guided focused ultrasound surgery. Cancer Res..

[31] Furusawa H., Namba K., Nakahara H., Tanaka C., Yasuda Y., Hirabara E., Imahariyama M., Komaki K. (2007). The evolving non-surgical ablation of breast cancer: MR guided focused ultrasound (MRgFUS). Breast Cancer.

[32] Wu F., Wang Z.B., Cao Y.D., Zhu X.Q., Zhu H., Chen W.Z., Zou J.Z. (2007). “Wide local ablation” of localized breast cancer using high intensity focused ultrasound. J. Surg. Oncol..

[33] Furusawa H., Namba K., Thomsen S., Akiyama F., Bendet A., Tanaka C., Yasuda Y., Nakahara H. (2006). Magnetic resonance–guided focused ultrasound surgery of breast cancer: Reliability and effectiveness. J. Am. Coll. Surg..

[34] Wu F., Wang Z.-B., Zhu H., Chen W.-Z., Zou J.-Z., Bai J., Li K.-Q., Jin C.-B., Xie F.-L., Su H.-B. (2005). Extracorporeal high intensity focused ultrasound treatment for patients with breast cancer. Breast Cancer Res. Treat..

[35] Zippel D.B., Papa M.Z. (2005). The use of MR imaging guided focused ultrasound in breast cancer patients; a preliminary phase one study and review. Breast Cancer.

[36] Wu F., Wang Z.-B., Cao Y.-D., Chen W., Bai J., Zou J., Zhu H. (2003). A randomised clinical trial of high-intensity focused ultrasound ablation for the treatment of patients with localised breast cancer. Br. J.Cancer.

[37] Gianfelice D., Khiat A., Amara M., Belblidia A., Boulanger Y. (2003). MR imaging–guided focused us ablation of breast cancer: Histopathologic assessment of effectiveness—Initial experience 1. Radiology.

[38] Hynynen K., Pomeroy O., Smith D.N., Huber P.E., McDannold N.J., Kettenbach J., Baum J., Singer S., Jolesz F.A. (2001). MR imaging-guided focused ultrasound surgery of fibroadenomas in the breast: A feasibility study 1. Radiology.

[39] Peek MC L., Wu F. (2018). High-intensity focused ultrasound in the treatment of breast tumours. Ecancermedicalscience.

[40] Ahmed H., Zacharakis E., Dudderidge T., Armitage J., Scott R., Calleary J., Illing R., Kirkham A., Freeman A., Ogden C. (2009). High-intensity-focused ultrasound in the treatment of primary prostate cancer: The first UK series. Br. J. Cancer.

[41] Chaussy C., Thuroff S. (2003). The status of high-intensity focused ultrasound in the treatment of localized prostate cancer and the impact of a combined resection. Curr. Urol. Rep..

[42] Beerlage H.P., Thuroff S., Debruyne F.M., Chaussy C., de la Rosette J.J. (1999). Transrectal high-intensity focused ultrasound using the Ablatherm device in the treatment of localized prostate carcinoma. Urology.

[43] Gelet A., Chapelon J.Y., Bouvier R., Souchon R., Pangaud C., Abdelrahim A.F., Cathignol D., Dubernard J.M. (1996). Treatment of prostate cancer with transrectal focused ultrasound: Early clinical experience. Eur. Urol..

[44] Chaussy C., Thuroff S. (2000). High-intensity focused ultrasound in prostate cancer: Results after 3 years. Mol. Urol..

[45] Diederich C.J., Wootton J., Prakash P., Salgaonkar V., Juang T., Scott S., Chen X., Cunha A., Pouliot J., Hsu I. (2011). Catheter-based ultrasound hyperthermia with HDR brachytherapy for treatment of locally advanced cancer of the prostate and cervix. Energy-based Treatment of Tissue and Assessment VI.

[46] Ripert T., Azémar M.-D., Ménard J., Bayoud Y., Messaoudi R., Duval F., Staerman F. (2010). Transrectal high-intensity focused ultrasound (HIFU) treatment of localized prostate cancer: Review of technical incidents and morbidity after 5 years of use. Prostate Cancer Prostatic Dis..

[47] Blana A., Walter B., Rogenhofer S., Wieland W.F. (2004). High-intensity focused ultrasound for the treatment of localized prostate cancer: 5-year experience. Urology.

[48] Chaussy C., Thuroff S., Rebillard X., Gelet A. (2005). Technology insight: High-intensity focused ultrasound for urologic cancers. Nat. Clin. Pract. Urol..

[49] Rebillard X., Gelet A., Davin J.L., Soulie M., Prapotnich D., Cathelineau X., Rozet F., Vallancien G. (2005). Transrectal high-intensity focused ultrasound in the treatment of localized prostate cancer. J. Endourol..

[50] Klingler H.C., Susani M., Seip R., Mauermann J., Sanghvi N., Marberger M.J. (2008). A novel approach to energy ablative therapy of small renal tumours: Laparoscopic high-intensity focused ultrasound. Eur. Urol..

[51] Illing R., Kennedy J., Wu F., Ter Haar G., Protheroe A., Friend P., Gleeson F., Cranston D., Phillips R., Middleton M. (2005). The safety and feasibility of extracorporeal high-intensity focused ultrasound (HIFU) for the treatment of liver and kidney tumours in a Western population. Br. J. Cancer.

[52] KÖHRMANN K.U., Michel M.S., Gaa J., Marlinghaus E., Alken P. (2002). High intensity focused ultrasound as noninvasive therapy for multilocal renal cell carcinoma: Case study and review of the literature. J. Urol..

[53] Wu F., Wang Z.-B., Chen W.-Z., Bai J., Zhu H., Qiao T.-Y. (2003). Preliminary experience using high intensity focused ultrasound for the treatment of patients with advanced stage renal malignancy. J. Urol..

[54] Arya M., Ahmed H.U., Scardino P., Emberton M. (2011). Interventional Techniques in Uro-Oncology.

[55] Napier K.J., Scheerer M., Misra S. (2014). Esophageal cancer: A Review of epidemiology, pathogenesis, staging workup and treatment modalities. World J. Gastrointest Oncol..

[56] Melodelima D., Prat F., Fritsch J., Theillere Y., Cathignol D. (2008). Treatment of esophageal tumors using high intensity intraluminal ultrasound: First clinical results. J. Transl. Med..

[57] He S., Wang G., Niu S., Yao B., Wang X. (2002). The noninvasive treatment of 251 cases of advanced pancreatic cancer with focused ultrasound surgery. Proceedings of the 2nd International Symposium on Therapeutic.

[58] Napoli A., Anzidei M., Marincola B.C., Brachetti G., Noce V., Boni F., Bertaccini L., Passariello R., Catalano C. (2013). MR Imaging–guided Focused Ultrasound for Treatment of Bone Metastasis. Radiographics.

[59] Xiong L.L., Hwang J.H., Huang X.B., Yao S.S., He C.J., Ge X.H., Ge H.Y., Wang X.F. (2009). Early clinical experience using high intensity focused ultrasound for palliation of inoperable pancreatic cancer. JOP.

[60] Izumi M., Ikeuchi M., Kawasaki M., Ushida T., Morio K., Namba H., Graven-Nielsen T., Ogawa Y., Tani T. (2013). MR-guided focused ultrasound for the novel and innovative management of osteoarthritic knee pain. BMC Musculoskelet. Disord..

[61] Moreno-Moraga J., Valero-Altes T., Riquelme A.M., Isarria-Marcosy M.I., de la Torre J.R. (2007). Body contouring by non-invasive transdermal focused ultrasound. Lasers Surg. Med..

[62] Zhao H., Yang G., Wang D., Yu X., Zhang Y., Zhu J., Ji Y., Zhong B., Zhao W., Yang Z. (2010). Concurrent gemcitabine and high-intensity focused ultrasound therapy in patients with locally advanced pancreatic cancer. Anti-Cancer Drugs.

[63] Lipsman N., Mainprize T.G., Schwartz M.L., Hynynen K., Lozano A.M. (2014). Intracranial applications of magnetic resonance-guided focused ultrasound. Neurotherapeutics.

[64] McDannold N., Clement G.T., Black P., Jolesz F., Hynynen K. (2010). Transcranial magnetic resonance imaging- guided focused ultrasound surgery of brain tumors: Initial findings in 3 patients. Neurosurgery.

[65] McDannold N., Vykhodtseva N., Jolesz F.A., Hynynen K. (2004). MRI investigation of the threshold for thermally induced blood-brain barrier disruption and brain tissue damage in the rabbit brain. Magn. Reson. Med..

[66] Cohen-Inbar O., Xu Z., Sheehan J.P. (2016). Focused ultrasound-aided immunomodulation in glioblastoma multiforme: A therapeutic concept. J. Ther. Ultrasound.

[67] Liberman B., Gianfelice D., Inbar Y., Beck A., Rabin T., Shabshin N., Chander G., Hengst S., Pfeffer R., Chechick A. (2009). Pain palliation in patients with bone metastases using MR-guided focused ultrasound surgery: A multicenter study. Ann. Surg. Oncol..

[68] Chen W., Zhu H., Zhang L., Li K., Su H., Jin C., Zhou K., Bai J., Wu F., Wang Z. (2010). Primary bone malignancy: Effective treatment with high-intensity focused ultrasound ablation 1. Radiology.

[69] Li C., Zhang W., Fan W., Huang J., Zhang F., Wu P. (2010). Noninvasive treatment of malignant bone tumors using high-intensity focused ultrasound. Cancer.

[70] Temple M.J., Waspe A.C., Amaral J.G., Napoli A., LeBlang S., Ghanouni P., Bucknor M.D., Campbell F., Drake J.M. (2016). Establishing a clinical service for the treatment of osteoid osteoma using magnetic resonance-guided focused ultrasound: Overview and guidelines. J. Ther. Ultrasound.

[71] Stewart E.A. (2001). Uterine fibroids. Lancet.

[72] LeBlang S.D., Hoctor K., Steinberg F.L. (2010). Leiomyoma shrinkage after MRI-guided focused ultrasound treatment: Report of 80 patients. AJR Am. J. Roentgenol..

[73] Stewart E.A., Rabinovici J., Tempany C.M., Inbar Y., Regan L., Gostout B. (2006). Clinical outcomes of focused ultrasound surgery for the treatment of uterine fibroids. Fertil. Steril..

[74] Lénárd Z.M., McDannold N.J., Fennessy F.M., Stewart E.A., Jolesz F.A., Hynynen K., Tempany C.M. (2008). Uterine Leiomyomas: MR Imaging–guided Focused Ultrasound Surgery—Imaging Predictors of Success 1. Radiology.

[75] Gorny K.R., Borah B.J., Brown D.L., Woodrum D.A., Stewart E.A., Hesley G.K. (2014). Incidence of Additional Treatments in Women Treated with MR-Guided Focused US for Symptomatic Uterine Fibroids: Review of 138 Patients with an Average Follow-up of 2.8 Years. J. Vasc. Interv. Radiol..

[76] Nau W.H., Diederich C.J., Simko J., Juang T., Jacoby A., Burdette E.C. (2007). Ultrasound Interstitial Thermal Therapy (USITT) for the Treatment of Uterine Myomas. Thermal Treatment of Tissue: Energy Delivery and Assessment IV.

[77] Rueff L.E., Raman S.S. (2013). Clinical and Technical Aspects of MR-Guided High Intensity Focused Ultrasound for Treatment of Symptomatic Uterine Fibroids. Semin. Interv. Radiol..

[78] Peek M.C., Ahmed M., Pinder S.E., Douek M. (2016). A review of ablative techniques in the treatment of breast fibroadenomata. J. Ther. Ultrasound.

[79] Lipsman N., Schwartz M.L., Huang Y., Lee L., Sankar T., Chapman M., Hynynen K., Lozano A.M. (2013). MR-guided focused ultrasound thalamotomy for essential tremor: A proof-of-concept study. Lancet Neurol..

[80] Elias W.J., Huss D., Voss T., Loomba J., Khaled M., Zadicario E., Frysinger R.C., Sperling S.A., Wylie S., Monteith S.J. (2013). A pilot study of focused ultrasound thalamotomy for essential tremor. N. Engl. J. Med..

[81] Jordão J.F., Thévenot E., Markham-Coultes K., Scarcelli T., Weng Y.-Q., Xhima K., O’Reilly M., Huang Y., McLaurin J., Hynynen K. (2013). Amyloid-β plaque reduction, endogenous antibody delivery and glial activation by brain-targeted, transcranial focused ultrasound. Exp. Neurol..

[82] Monteith S.J., Kassell N.F., Goren O., Harnof S. (2013). Transcranial MR-guided focused ultrasound sonothrombolysis in the treatment of intracerebral hemorrhage. Neurosurg. Focus.

[83] Martin E., Jeanmonod D., Morel A., Zadicario E., Werner B. (2009). High-intensity focused ultrasound for noninvasive functional neurosurgery. Ann. Neurol..

[84] Jeanmonod D., Werner B., Morel A., Michels L., Zadicario E., Schiff G., Martin E. (2012). Transcranial magnetic resonance imaging-guided focused ultrasound: Noninvasive central lateral thalamotomy for chronic neuropathic pain. Neurosurg. Focus.

[85] Burgess A., Huang Y., Waspe A.C., Ganguly M., Goertz D.E., Hynynen K. (2012). High-intensity focused ultrasound (HIFU) for dissolution of clots in a rabbit model of embolic stroke. PLoS ONE.

[86] Hölscher T., Ahadi G., Fisher D., Zadicario E., Voie A. (2013). MR-guided focused ultrasound for acute stroke a rabbit model. Stroke.

[87] Wright C., Hynynen K., Goertz D. (2012). In vitro and in vivo high intensity focused ultrasound thrombolysis. Invest Radiol..

[88] Alkins R., Huang Y., Pajek D., Hynynen K. (2013). Cavitation-based third ventriculostomy using MRI-guided focused ultrasound: Laboratory investigation. J. Neurosurg..

[89] Chang W.S., Jung H.H., Kweon E.J., Zadicario E., Rachmilevitch I., Chang J.W. (2015). Unilateral magnetic resonance guided focused ultrasound thalamotomy for essential tremor: Practices and clinicoradiological outcomes. J. Neurol. Neurosurg. Psychiatry.

[90] Lozano A.M., Lipsman N. (2013). Probing and regulating dysfunctional circuits using deep brain stimulation. Neuron.

[91] Magara A., Bühler R., Moser D., Kowalski M., Pourtehrani P., Jeanmonod D. (2014). First experience with MR-guided focused ultrasound in the treatment of Parkinson’s disease. J. Ther. Ultrasound.

[92] Cetas J.S., Saedi T., Burchiel K.J. (2008). Destructive procedures for the treatment of nonmalignant pain: A structured literature review. J. Neurosurg..

[93] Sullivan L.D., McLoughlin M.G., Goldenberg L.G., Gleave M.E., Marich K.W. (1997). Early experience with high-intensity focused ultrasound for the treatment of benign prostatic hypertrophy. Br. J. Urol..

[94] Madersbacher S., Schatzl G., Djavan B., Stulnig T., Marberger M. (2000). Long-term outcome of transrectal high- intensity focused ultrasound therapy for benign prostatic hyperplasia. Eur. Urol..

[95] Bergenfelz A., Jansson S., Kristoffersson A., Mårtensson H., Reihnér E., Wallin G., Lausen I. (2008). Complications to thyroid surgery: Results as reported in a database from a multicenter audit comprising 3,660 patients. Langenbecks Arch. Surg..

[96] Kovatcheva R.D., Vlahov J.D., Stoinov J.I., Zaletel K. (2015). Benign Solid Thyroid Nodules: US-guided High-Intensity Focused Ultrasound Ablation—Initial Clinical Outcomes. Radiology.

[97] Esnault O., Franc B., Ménégaux F., Rouxel A., De Kerviler E., Bourrier P., Lacoste F., Chapelon J.Y., Leenhardt L. (2011). High-intensity focused ultrasound ablation of thyroid nodules: First human feasibility study. Thyroid.

[98] Korkusuz H., Fehre N., Sennert M., Happel C., Grünwald F. (2015). Volume reduction of benign thyroid nodules 3 months after a single treatment with high-intensity focused ultrasound (HIFU). J. Ther. Ultrasound.

[99] Leenhardt L., Rouxel A., Menegaux F., Esnault O. (2013). An open-label, randomized, controlled study of the effectiveness and safety of a high intensity focused ultrasound device compared with observation in patients with non-malignant cold thyroid nodules. Endocr. Abstr..

[100] Korkusuz H., Fehre N., Sennert M., Happel C., Grünwald F. (2014). Early assessment of high-intensity focused ultrasound treatment of benign thyroid nodules by scintigraphic means. J. Ther. Ultrasound.

[101] Kovatcheva R.D., Zaletel K. (2017). High-intensity focused ultrasound for thyroid nodule ablation: The evidence to date. Rep. Med Imaging.

[102] Kovatcheva R.D., Vlahov J.D., Stoinov J.I., Zaletel K. (2015). The effect of one and two sessions of US-guided high-intensity focused ultrasound (HIFU) treatment on thyroid nodule volume and thyroid function. Thyroid.

[103] Gliklich R.E., White W.M., Slayton M.H., Barthe P.G., Makin I.R.S. (2007). Clinical pilot study of intense ultrasound therapy to deep dermal facial skin and subcutaneous tissues. Arch. Facial Plast. Surg..

[104] Wu F., Wang Z.-B., Chen W.-Z., Zou J.-Z., Bai J., Zhu H., Li K.-Q., Xie F.-L., Jin C.-B., Su H.-B. (2004). Extracorporeal focused ultrasound surgery for treatment of human solid carcinomas: Early Chinese clinical experience. Ultrasound Med. Biol..

[105] Napoli A., Anzidei M., De Nunzio C., Cartocci G., Panebianco V., De Dominicis C., Catalano C., Petrucci F., Leonardo C. (2013). Real-time magnetic resonance-guided high-intensity focused ultrasound focal therapy for localised prostate cancer: Preliminary experience. Eur. Urol..

[106] Okada A., Murakami T., Mikami K., Onishi H., Tanigawa N., Marukawa T., Nakamura H. (2006). A case of hepatocellular carcinoma treated by MR-guided focused ultrasound ablation with respiratory gating. Magn. Reson. Med Sci..

[107] Zhou Y. (2015). Principles and Applications of Therapeutic Ultrasound in Healthcare.

[108] Vykhodtseva N., McDannold N., Hynynen K. (2008). Progress and problems in the application of focused ultrasound for blood-brain barrier disruption. Ultrasonics.

[109] Wei K.-C., Chu P.-C., Wang H.-Y.J., Huang C.-Y., Chen P.-Y., Tsai H.-C., Lu Y.-J., Lee P.-Y., Tseng I.-C., Feng L.-Y. (2013). Focused ultrasound-induced blood–brain barrier opening to enhance temozolomide delivery for glioblastoma treatment: A preclinical study. PLoS ONE.

[110] Thevenot E., Jordao J.F., O’Reilly M.A., Markham K., Weng Y.Q., Foust K.D., Kaspar B.K., Hynynen K., Aubert I. (2012). Targeted delivery of self-complementary adeno-associated virus serotype 9 to the brain, using magnetic resonance imaging-guided focused ultrasound. Hum. Gene Ther..

[111] Samiotaki G., Konofagou E.E. (2013). Dependence of the reversibility of focused- ultrasound-induced blood-brain barrier opening on pressure and pulse length in vivo. IEEE Trans. Ultrason. Ferroelectr. Freq. Control.

[112] Bonow R.H., Silber J.R., Enzmann D.R., Beauchamp N.J., Ellenbogen R.G., Mourad P.D. (2016). Towards use of MRI-guided ultrasound for treating cerebral vasospasm. J. Ther. Ultrasound.

[113] Rieck B., Bates D., Zhang K., Escott N., Mougenot C., Pichardo S., Curiel L. (2014). Focused ultrasound treatment of abscesses induced by methicillin resistant Staphylococcus aureus: Feasibility study in a mouse model. Med. Phys..

[114] Smith N.B. (2008). Applications of ultrasonic skin permeation in transdermal drug delivery. Expert Opin. Drug Deliv..

[115] Pitt W.G., Husseini G.A., Staples B.J. (2004). Ultrasonic drug delivery-a general review. Expert Opin. Drug Deliv..

[116] Fan C.-H., Ting C.-Y., Lin C.Y., Chan H.-L., Chang Y.-C., Chen Y.-Y., Liu H.-L., Yeh C.-K. (2016). Noninvasive, targeted, and non-viral ultrasound-mediated GDNF-plasmid delivery for treatment of Parkinson’s disease. Sci. Rep..

[117] Meng Y., Solomon B., Boutet A., Llinas M., Scantlebury N., Huang Y., Hynynen K., Hamani C., Fasano A., Lozano A.M. (2018). Magnetic resonance-guided focused ultrasound thalamotomy for treatment of essential tremor: A 2-year outcome study. Mov. Disord..

[118] Zaaroor M., Sinai A., Goldsher D., Eran A., Nassar M., Schlesinger I. (2018). Magnetic resonance-guided focused ultrasound thalamotomy for tremor: A report of 30 Parkinson’s disease and essential tremor cases. J. Neurosurg..

[119] Iacopino D.G., Gagliardo C., Giugno A., Giammalva G.R., Napoli A., Maugeri R., Graziano F., Valentino F., Cosentino G., D’Amelio M. (2018). Preliminary experience with a transcranial magnetic resonance-guided focused ultrasound surgery system integrated with a 1.5-T MRI unit in a series of patients with essential tremor and Parkinson’s disease. Neurosurg. Focus.

[120] Kubanek J. (2018). Neuromodulation with transcranial focused ultrasound. Neurosurg. Focus.

[121] Jung H.H., Chang W.S., Kim S.J., Kim C.-H., Chang J.W. (2018). The Potential Usefulness of Magnetic Resonance Guided Focused Ultrasound for Obsessive Compulsive Disorders. J. Korean Neurosurg. Soc..

